# Estimation of Alkaline Phosphatase Activity in Ovarian Stem Cells With and Without the Use of Additives Replacing Fetal Bovine Serum

**DOI:** 10.7759/cureus.88282

**Published:** 2025-07-19

**Authors:** Sindhuja Srinivasan, Sanjeeva Reddy, Alan Mathew Punnoose, Madan Kalagara, Mubeena S, Paramesh Vishwanathan

**Affiliations:** 1 Reproductive Medicine and Surgery, Sri Ramachandra Institute of Higher Education and Research, chennai, IND; 2 Reproductive Medicine and Surgery, Sri Ramachandra Institute of Higher Education and Research, Chennai, IND; 3 Stem Cell and Regenerative Biology Laboratory, Sri Ramachandra Institute of Higher Education and Research, Chennai, IND; 4 Pathology, Vijaya Medical Centre, Vishakapatnam, IND

**Keywords:** alkaline phosphatase, fetal bovine serum, follicular fluid, ovarian stem cells, pluripotency, pooled platelet lysate, umbilical cord blood

## Abstract

Ovarian stem cells (OSCs) are a promising tool for reproductive regeneration, but ethical concerns surrounding fetal bovine serum (FBS) use have prompted the search for viable alternatives. This study estimates alkaline phosphatase (ALP) activity-a pluripotency marker-in two OSC lines (OTC-5, OTC-7) cultured with FBS and with potential replacements: cord blood serum (CB), follicular fluid (FF), and pooled platelet lysate (PP). ALP activity was quantified using a colorimetric p-nitrophenyl phosphate assay. Results show that FF and PP can maintain or slightly improve ALP activity compared to controls, supporting their feasibility as xeno-free culture additives for OSCs.

## Introduction

Ovarian stem cells (OSCs) are emerging as a novel source for fertility preservation, reproductive rejuvenation, and regenerative medicine as stated in recent studies [1,2]. According to Bhartiya et al., these rare germline stem cells, located within the ovarian surface epithelium and cortical tissue, have been shown to possess mitotic activity and the potential to generate oocytes even in adult ovaries [3]. As a result, OSCs are attracting significant interest for their possible role in overcoming age-related infertility, premature ovarian failure, and other reproductive disorders.

However, the successful in vitro expansion and maintenance of OSCs remains a major challenge. Conventional culture methods largely depend on fetal bovine serum (FBS) as a nutrient-rich supplement providing essential growth factors, hormones, and adhesion molecules [4]. Despite its widespread use, FBS presents several limitations including ethical concerns related to animal welfare, the risk of transmitting zoonotic pathogens, and significant batch-to-batch variability identified by Burnouf et al. which in turn can affect experimental reproducibility [5]. In the context of translational research and clinical applications, these drawbacks necessitate the development of safer, xeno-free, and ethically acceptable alternatives.

Recently, various human-derived supplements such as cord blood (CB) serum, follicular fluid (FF), and human platelet lysate (hPL) [6] have been explored by several groups to replace FBS in stem cell culture. These biological fluids are rich in bioactive molecules and growth factors that may support the proliferation and maintenance of stem cells under more clinically relevant conditions. However, their impact on the fundamental characteristics of OSCs, including stemness and differentiation potential, requires systematic evaluation.

Alkaline phosphatase (ALP) is widely recognized as a functional biomarker of pluripotency and undifferentiated status in various stem cell types, including embryonic, germline, and mesenchymal stem cells shown in several studies [7,8]. High ALP activity indicates active self-renewal pathways and an undifferentiated state, whereas reduced ALP levels often accompany spontaneous differentiation or suboptimal culture conditions. Therefore, ALP activity serves as a simple but reliable indicator of the ability of different culture additives to maintain OSC stemness.

In this study, we aimed to compare the ALP activity of OSCs cultured under standard conditions with FBS to those cultured with human-derived additives such as CB serum, FF, and pooled platelet lysate (PP). By estimating and analyzing ALP activity under these conditions, we hope to identify viable and reproducible alternatives to FBS that support the clinical translation of OSC-based therapies.

## Materials and methods

Procurement and processing of ovarian tissue

Ovarian cortical tissue was obtained from two patients aged 43 years (Sample 1 labeled OTC-5) and 50 years (Sample 2 labeled OTC-7) undergoing oophorectomy at our University Hospital after written informed consent (IEC Approval number: IEC-NI/18/jan/63/12). Indication for oophorectomy in the patients was the presence of unilateral ovarian cyst. The ovarian tissue was collected from the side which was not affected by ovarian cyst. Histopathological investigation of both affected and unaffected ovaries sent for sampling were non-malignant in both the cases. The stem cell lines isolated by the below mentioned protocol was labeled as OTC-5 and OTC-7 from the first and second patient respectively.

Ovarian tissue was brought to the stem cell lab in cold phosphate buffered saline(PBS). After thorough rinsing, it was mechanically dissected and exposed to collagenase and hyaluronidase (80 IU/ml) overnight in a roller mixer at 37°C in a carbon dioxide incubator. The cells were then left for culture in AlphaMEM with antibiotic and 10% FBS, in 100 mm culture grade dish. Cells were serially passaged, the cells in Passage 3 were used for the cell treatment and assessment of ALP.

Preparation of pooled human serum from cord blood

After obtaining proper informed consent, blood was collected from the umbilical cord which will otherwise be discarded. The CB was centrifuged at 300G for 15 minutes to obtain sera which was frozen. When adequate sample was obtained (n=10), the sera was thawed, pooled and filtered through a 0.22 µ filter. The obtained composition was used as a serum supplement. They were frozen in a −80°C freezer and stored until use. At the time of usage, the serum was thawed to 37°C in a water bath and added to media

Preparation of pooled human follicular fluid

After proper informed consent, FF which will otherwise be discarded was collected from patients undergoing oocyte retrieval as a part of their in-vitro fertilization (IVF) treatment in the department of reproductive medicine and surgery, Sri Ramachandra University. The fluid was treated with heparin and centrifuged at 1500 rpm to discard red blood cells- the supernatant fluid alone was used as supplement. These supplements were pooled, filtered and heat inactivated at 560C for 30 minutes in a water bath. They were frozen at −80°C freezer and stored until use. At the time of usage, the fluid was thawed to 37°C in a water bath and added to media.

Preparation of pooled platelet lysate

The platelets which are usually discarded from the blood bank after the time frame beyond which they cannot be used(approximately 7 days), was collected and pooled. At the time of use, they were processed by self-fractionation by repeated freeze thaw cycles (−80°C to 37°C using a freezer and water bath) following which centrifugation was done at 4000 g for 15 minutes and filtered through a 0.22 µm filter.

Cell culture

OTC-5, OTC-7 cells were maintained in stem cell medium supplemented with or without 10% FBS, 100 µg/ml penicillin and 100 µg/ml streptomycin, and maintained under an atmosphere of 5% CO_2_ at 37°C.

Cells treatment

OTC-5, OTC-7 cells were seeded into a six-well tissue culture dish and allowed to grow to 90% confluency in a complete medium. The cells were treated with 10% of CB, FF, PP samples in a stem cell basal medium with FBS as control and incubated for 7 days. The timeline of 7 days was decided based on previous colony-forming unit analysis of these cell lines which showed maximum potential to maintain cell line without differentiation compared to 14 days and 21 days. All the assessments were performed in triplicates to avoid experimental errors.

Procedure

The measurement of ALP activity in the test sample treated cell culture supernatant was carried out using an ALP assay kit, according to the manufacturer’s instructions (Sigma, USA). 980 μl of reaction buffer was transferred into one cuvette (blank). Then 960 μl of reaction buffer was pipetted into additional cuvettes (one for each test or enzyme control). 20 μl of 0.67M pNPP solution was added to each cuvette (test and control). The cuvettes were equilibrated to 37°C. The test sample 20 μl was added to each test cuvette. 20 μl of the diluted ALP solution was added to the enzyme control well. This was immediately mixed by inversion and recorded the increase in A405 nm for 5 minutes. The maximum linear rate (∆A405nm/minute) for the test, blank and control was observed. The ALP levels based on optical density was assessed using the formula:

Units/mL= (∆A405 nm/min Test - ∆A405 nm/min Blank) (df) (VF)/(18.5)(VE)

where,

df: dilution factor (100)

VF: volume (in ml) of assay (2 ml)

18.5: Millimolar extinction coefficient of pNPP at 405 nm

VE: Volume (in ml) of sample solution used (0.02 ml)

## Results

Optical density measurements

The optical density (OD) values at 405 nm for each treatment condition are summarized in Table 1. For the OTC-5 cell line, cells cultured with FF supplementation demonstrated the highest OD (0.728), followed by platelet lysate (0.664) and CB serum (0.648), all compared to the FBS control (0.631). A similar trend was observed for the OCT-7 line, where FF (0.672) and PP (0.659) yielded higher OD readings than the control (0.632). In contrast, CB serum supplementation showed a slightly lower OD in OCT-7 (0.587), suggesting a modest reduction in ALP substrate conversion compared to FBS. The OD of the control, OTC5 and OTC7 was measured in triplicates. The OD values are displayed in Table 1.

**Table 1:** Optical density value in triplicates for OTC5 and OTC7 with and without follicular fluid, cord blood, and pooled platelet lysate as supplement

Test Samples	Mean OD Values at 405 nm
OCT-5 Control	0.631
OCT-5 Cord Blood (CB)	0.648
OCT-5 Follicular Fluid (FF)	0.728
OCT-5 Pooled Platelet Lysate (PP)	0.664
OCT-7 Control	0.632
OCT-7 Cord Blood (CB)	0.587
OCT-7 Follicular Fluid (FF)	0.672
OCT-7 Pooled Platelet Lysate (PP)	0.659

Calculated alkaline phosphatase (ALP) activity

The corresponding calculated ALP activities (expressed in units/ml) are presented in Table 2. In the OTC-5 line, FF supplementation resulted in the highest ALP activity (0.139 U/ml), followed by platelet lysate (0.126 U/ml) and CB serum (0.123 U/ml), compared to the FBS control (0.120 U/ml). Notably, FF supplementation increased ALP activity by approximately 15.8% relative to control.

**Table 2:** Alkaline phosphatase levels in OTC5 and OTC7 with and without use of follicular fluid, cord blood, and platelet lysate supplements.

Test Samples	ALP Units/ml
OCT-5 Control	0.12
OCT-5 Cord blood (CB)	0.123
OCT-5 Follicular fluid (FF)	0.139
OCT-5 Pooled platelet lysate (PP)	0.126
OCT-7 Control	0.12
OCT-7 Cord blood (CB)	0.111
OCT-7 Follicular fluid (FF)	0.128
OCT-7 Pooled platelet lysate (PP)	0.125

For the OTC-7 cell line, a similar pattern was observed: FF (0.128 U/ml) and platelet lysate (0.125 U/ml) maintained ALP activity close to or slightly higher than the control (0.120 U/ml). However, CB serum supplementation showed a small decrease in ALP activity (0.111 U/ml) in OCT-7 cells compared to control, indicating possible cell-line specific variation in response to CB.

Across both cell lines, FF consistently supported the highest ALP activity, suggesting a stronger ability to preserve OSC stemness in vitro. PP also maintained ALP activity comparable to FBS. CB serum was effective for the OCT-5 line but demonstrated slightly reduced ALP activity in the OCT-7 line.

No morphological changes indicative of overt differentiation were observed during the 7-day culture period in any of the treatment groups. All treatments were well tolerated by the OSCs, and the cells maintained typical stem-like morphology and confluency under all conditions.

Together, these findings confirm that FF and platelet lysate are promising xeno-free alternatives to FBS for maintaining ALP activity in OSCs under in vitro conditions.

## Discussion

The present data suggest that human FF and PP can sustain ALP activity in OSCs, suggesting that these human-derived additives are capable of maintaining the undifferentiated state in vitro without relying on animal-derived serum. This is a significant finding, as it demonstrates the practical potential of developing xeno-free culture systems for OSC expansion - an essential step toward safe and ethical clinical applications as previously suggested by Rauch et al. and Bournof et al. [5,6].

The observed ALP activity levels in cultures supplemented with FF were slightly higher than the standard FBS control, indicating that FF provides a microenvironment that may closely mimic the native ovarian niche. Jafarpour et al. and Yan et al. have demonstrated that FF is naturally rich in growth factors, hormones, and cytokines that promote oocyte maturation and folliculogenesis, which could explain its supportive effect on OSC stemness markers in vitro [9,10]. Similar trends have been reported in oocyte and granulosa cell cultures where FF enhanced cell viability and developmental competence.

PP also maintained ALP activity comparable to FBS-containing media. Platelet lysate is increasingly recognized as a safe and effective alternative to FBS for expanding mesenchymal and hematopoietic stem cells, as it provides a broad spectrum of growth factors such as PDGF, TGF-β, and epidermal growth factor (EGF). Its success here with OSCs highlights its versatility and underlines its potential as a standardized supplement for clinical-grade stem cell production.

Interestingly, CB serum resulted in slightly lower ALP activity in the OCT-7 line compared to FBS, although it maintained comparable activity in OCT-5. This suggests that the effectiveness of certain human-derived supplements may vary between OSC lines, possibly due to differences in receptor expression, signaling pathways, or baseline metabolic activity. Such cell-line specific responses underline the importance of careful optimization and standardization when developing universal, xeno-free culture protocols.

Collectively, these findings support the feasibility of replacing FBS with ethically acceptable and clinically safer human-derived additives. However, ALP activity alone does not fully capture the functional potential of OSCs. Therefore, further investigations should focus on assessing long-term proliferation rates, karyotypic stability, and the cells’ capacity to differentiate into functional oocytes or other relevant lineages. Additionally, the scalability, donor variability, and quality control of these human supplements need to be addressed to ensure reproducibility and compliance with regulatory requirements for future clinical applications.

## Conclusions

This study demonstrates that human-derived additives, particularly FF and PP, can effectively maintain ALP activity in OSCs, indicating that these cells can retain their undifferentiated state in vitro without relying on animal-derived FBS. Furthermore, using components such as FF and platelet lysate may better mimic the OSC’s natural microenvironment, supporting their proliferation and functional characteristics in vitro.

However, ALP activity alone is not sufficient to confirm the complete preservation of OSC stemness and functional potential. Therefore, additional studies should further validate these findings by evaluating long-term expansion, genomic stability, and differentiation capacity into oocyte-like structures or other germline lineages. Moreover, standardizing the preparation, quality control, and scalability of human-derived supplements will be critical for their broader adoption in clinical-grade OSC culture systems. In summary, this work contributes valuable preliminary evidence supporting the feasibility of xeno-free alternatives for OSC culture and highlights a promising direction for developing ethically sound, reproducible, and translationally relevant protocols for regenerative reproductive medicine.
